# The relationship between physical activity and mental health among university students: a chain mediation effect of self-efficacy and emotion regulation

**DOI:** 10.3389/fpsyg.2025.1681753

**Published:** 2025-11-19

**Authors:** Jingshuo Wang, Qingjie Chen, Jie Meng, Junyang Wei, Chunhui Wang, Chunwei Hou

**Affiliations:** 1 School of Sports and Health, Linyi University, Linyi, China; 2 Network and Information Center, Qingdao Institute of Technology, Qingdao, China; 3 Department of Basic Courses, Peoples Liberation Army Engineering University - Shijiazhuang Campus, Shijiazhuang, China

**Keywords:** physical activity, mental health, self-efficacy, emotion regulation, COR

## Abstract

**Background:**

University students are increasingly vulnerable to mental health challenges, highlighting the need for targeted and theory-driven interventions. Physical activity has been widely recognized for its psychological benefits, yet the underlying psychological mechanisms remain insufficiently explored. Guided by the Conservation of Resources (COR) theory, this study investigates how physical activity influences mental health among university students through the chain-mediating roles of self-efficacy and emotion regulation.

**Methods:**

A cross-sectional survey was conducted among 1,395 university students in China. Validated instruments were used to assess physical activity, self-efficacy, emotion regulation, and mental health. Statistical analyses including descriptive statistics, correlation analysis, and mediation modeling were performed using SPSS 26.0 and PROCESS macro (Model 6) with 5,000 bootstrap samples.

**Results:**

The results showed significant correlations among the variables. Physical activity, as a positive resource investment behavior, significantly enhances self-efficacy and emotion regulation. These psychological resources work together to alleviate psychological distress. Chain mediation analysis indicated that self-efficacy and emotion regulation play a chain mediating role in the relationship between physical activity and mental health.

**Conclusion:**

This study advances our understanding of the psychological pathways linking physical activity to mental health by revealing a dual-step mediation mechanism. The findings highlight the importance of enhancing self-efficacy and emotional regulation in university-based mental health interventions. Integrating physical activity with psychological skill-building may offer an effective approach to promoting mental well-being in higher education settings.

## 1 Introduction

University students are at a critical stage of psychological adaptation and self-development, a period essential for both academic achievement and the cultivation of psychological competence and social functioning. Recent surveys have shown a steady increase in psychological stress, emotional disorders, and adjustment difficulties among university students (Sun et al., 2022). Reports further indicate that mental health risks in this population now exceed the societal average, with anxiety and depression being particularly prevalent (Li et al., 2021). Given this widespread psychological distress, there is an urgent need for more comprehensive and systematic approaches to promote mental health among university students.

China’s rapid expansion of higher education, coupled with intense academic demands and competitive employment pressures, has made the mental health of Chinese university students an especially salient concern. This population frequently experiences migration-related adaptation, academic overload, and limited access to psychological support services. Focusing on this group not only reflects the distinctive sociocultural features of China’s higher education system but also generates insights that can inform culturally sensitive mental health interventions and policy development in other collectivist societies. In recent years, physical activity has received growing recognition as an effective means of improving mental health. Systematic reviews and meta-analyses have confirmed that regular physical exercise can alleviate stress, enhance mood, and promote psychological well-being (Mikkelsen et al., 2017). However, the specific psychological mechanisms through which physical activity influences mental health remain a subject of debate. Although previous studies have proposed several pathways, the interplay of multiple mediating factors has not been thoroughly investigated and requires further empirical validation.

The Conservation of Resources (COR) theory provides a robust framework for explaining the complex relationship between physical activity and mental health (Hobfoll, 1989). This theory posits that individuals cope with life stress by acquiring, maintaining, and expanding various resources—emotional, cognitive, and social. The preservation and replenishment of these resources strengthen resilience and psychological adaptability, thereby buffering individuals against negative emotional outcomes (Hobfoll, 2001). Once acquired, resources can reinforce one another, creating a resource-cluster effect that produces long-term psychological benefits (Hobfoll, 2011). Guided by the COR theory, this study conceptualizes physical activity as a form of proactive resource investment that can initiate gain spirals. Within this framework, self-efficacy functions as a cognitive resource, while emotion regulation operates as an emotional resource that helps individuals restore affective balance. COR theory further suggests that cognitive resources such as self-efficacy can enhance emotional resources like emotion regulation, forming cross-type resource chains that strengthen psychological resilience and reduce distress.

University students face multiple adaptation challenges, making proactive resource investment and accumulation particularly critical for maintaining mental health. Empirical studies have shown that both emotion regulation and self-efficacy are strongly associated with mental health (Krifa et al., 2022; Zhou et al., 2021). However, there remains limited systematic evidence on how physical activity influences mental health through these psychological resources via multiple mediating pathways. To address this gap, the present study integrates self-efficacy and emotion regulation as key psychological resources that may independently or sequentially influence mental health. Based on the COR framework, a chain mediation model is proposed to examine how physical activity affects the mental health of university students. This study aims to provide both theoretical and empirical evidence for the scientific design of mental health promotion strategies and to offer new perspectives for psychological regulation in higher education.

### 1.1 Physical exercise in relation to self-efficacy and emotion regulation

In recent years, the facilitative role of physical activity in fostering positive psychological resources has received growing scholarly attention. Beyond improving physical fitness, physical exercise serves as a form of psychological resource investment that is closely linked to both self-efficacy and emotion regulation ability. Self-efficacy, a core construct of social cognitive theory, refers to an individual’s belief in their capability to organize and execute the actions required to achieve specific goals (Locke, 1997). Numerous empirical studies have demonstrated that regular participation in physical exercise significantly enhances university students’ self-efficacy (Tikac et al., 2022). Some research further suggests that physical activity may influence self-efficacy indirectly through the development of psychological resilience (Li et al., 2024). Based on a large-scale survey of Chinese university students, Mu et al. found that physical activity positively predicted general self-efficacy, with self-efficacy fully mediating the relationship between physical activity and emotion regulation ability (Mu F zheng et al., 2024). Students who engage in regular physical activity tend to exhibit greater confidence when facing challenges and are more likely to adopt stable and adaptive emotional coping strategies. Regarding emotion regulation, physical exercise also exerts a beneficial effect. Sheng et al. reported that physical activity not only directly reduces anxiety but also indirectly improves psychological states by enhancing individuals’ cognitive reappraisal abilities (Sheng et al., 2024). Physical activity fosters a stronger sense of self-control, enabling individuals to manage emotional fluctuations more effectively in complex life situations. Some researchers have proposed that cortical thickness plays an important role in the relationship between physical activity and emotion regulation among university students (Wu et al., 2022). Evidence also indicates that physical activity contributes to emotion regulation in diverse populations, including children and adults with chronic illnesses (Tse, 2020; Sadeghi Bahmani et al., 2020).

Overall, physical activity, self-efficacy, and emotion regulation jointly constitute a resource accumulation system. As an active form of resource investment, physical exercise may enhance emotion regulation indirectly by strengthening self-efficacy beliefs. This enhancement, in turn, provides internal support for managing environmental stress and maintaining mental health.

### 1.2 Mental health in relation to self-efficacy and emotion regulation ability

As a multidimensional construct reflecting individuals’ adaptability to stress, potential realization, and social functioning, mental health plays a crucial role in the development and adjustment of university students. Recent research has increasingly examined how positive psychological resources—particularly self-efficacy and emotion regulation—contribute to mental health. Self-efficacy has been widely identified as a key psychological determinant of mental health (White et al., 2024). It indirectly shapes psychological well-being by influencing individuals’ confidence, persistence, and perceived control when encountering challenging situations (Locke, 1997). Empirical evidence indicates a significant negative association between self-efficacy and depression among university students (Yang et al., 2022). Students with higher levels of general self-efficacy tend to report fewer psychological symptoms, especially under conditions such as test anxiety or interpersonal conflict, where self-efficacy functions as a protective factor (Kristensen et al., 2023). In parallel, emotion regulation represents a fundamental psychological mechanism for maintaining mental health. Individuals with stronger emotion regulation skills are better able to manage negative affect and cope with stress, thereby reducing vulnerability to psychological problems. According to Gross’s process model of emotion regulation, regulatory strategies are central components of cognitive-affective processing that directly influence mental health outcomes (Gross, 1998). Moreover, emotion regulation has been shown to mediate the association between physical activity and mental health (Tang et al., 2022). Self-efficacy and emotion regulation are interrelated. Individuals with higher self-efficacy are more likely to adopt adaptive strategies, display greater cognitive reappraisal ability, and show higher acceptance of emotional experiences (Ramos-Cejudo et al., 2024). Deficits in emotion regulation, by contrast, are closely associated with various mental disorders such as anxiety and depression. Importantly, emotion-regulation self-efficacy—the belief in one’s ability to manage emotions effectively—has been recognized as a crucial predictor of therapeutic improvement and an effective target in psychological interventions (Bardeen and Fergus, 2020; Macrynikola et al., 2024).

Together, these findings suggest that self-efficacy and emotion regulation jointly contribute to mental health through a capacity accumulation process, in which cognitive confidence and emotional competence reinforce one another. This perspective provides theoretical and empirical support for examining their serial mediating role in psychological adjustment mechanisms among university students.

### 1.3 The relationship between self-efficacy and emotion regulation ability

Self-efficacy not only directly predicts mental health but also serves as a key cognitive–motivational foundation for emotion regulation. Within the framework of social cognitive theory, self-efficacy represents an individual’s belief in their capacity to organize and execute actions required to manage emotional challenges. It determines how effectively people mobilize motivation, sustain effort, and adopt adaptive coping strategies in stressful contexts (Albert, 2012). Individuals with stronger self-efficacy perceive emotional stressors as controllable, which facilitates the use of constructive regulation strategies such as cognitive reappraisal and problem-focused coping (Ryan et al., 2019). This theoretical foundation suggests that self-efficacy provides the psychological energy and confidence necessary to initiate and maintain effective emotion regulation processes. Empirical studies have consistently confirmed this mechanism. Zhou et al. (2021) found that higher self-efficacy predicted lower mental health problems through adaptive cognitive and emotional processes during the COVID-19 pandemic, demonstrating its importance for emotional management under prolonged stress. Similarly, Li and Quan (2025) showed that regulatory emotional self-efficacy indirectly reduced adverse mental health outcomes via enhanced resilience among Chinese university students, providing strong support for the mediating role of emotion regulation in the self-efficacy–mental health link. In addition, Lande et al. (2023) demonstrated that adolescents with higher general self-efficacy exhibited both stronger neurophysiological and self-reported indicators of emotion regulation ability, suggesting that self-efficacy contributes to emotional control at multiple levels. Di Giunta et al. (2022) further confirmed that emotion-regulation self-efficacy mediates the relationship between parenting quality and adolescents’ mental health, underscoring its developmental significance. In recent years, empirical work has begun to provide additional support for the self-efficacy → emotion regulation → mental health pathway. Ramos-Cejudo et al. (2024) found that self-efficacy for cognitive reappraisal is associated with more frequent use of adaptive emotion regulation strategies and lower levels of affective symptoms across cross-sectional and longitudinal samples. Xu and Xu (2025) also reported that self-efficacy predicted better emotion regulation and psychological resilience. While direct evidence is scarcer, these findings align with the idea that self-efficacy may strengthen emotional regulation capacity, supporting the Conservation of Resources (COR) perspective in which cognitive resource gains bolster emotional stability and preserve psychological energy.

Overall, both theoretical and empirical evidence suggest that self-efficacy functions as a cognitive–motivational resource that facilitates emotion regulation by promoting confidence, persistence, and strategic flexibility. University students with higher levels of self-efficacy are more capable of maintaining composure under stress, applying adaptive emotion-regulation strategies, and recovering from negative affect.

### 1.4 Theoretical framework: a COR-based serial mediation

The Conservation of Resources (COR) theory proposes that individuals strive to acquire, preserve, and expand valuable resources, and that mental health improves when such resources are accumulated rather than depleted. Recent refinements of the theory highlight the dynamics of resource caravans and gain–loss cycles, through which resources interact, cluster, and reinforce one another over time. From this perspective, mental health is maintained through continual resource acquisition and accumulation, whereas resource depletion leads to maladaptive outcomes. Empirical evidence from the COVID-19 pandemic supports this view, showing that individuals with greater resource reserves report better mental health and resilience, consistent with COR’s central claim that gain spirals serve as protective mechanisms under prolonged stress (Egozi Farkash et al., 2022). Within this framework, physical activity can be viewed as a proactive investment of behavioral resources that initiates gain spirals. Through repeated mastery experiences and self-regulation, physical activity builds personal cognitive resources—most notably self-efficacy—and simultaneously generates social and emotional assets. Large-scale studies among university students have demonstrated that higher levels of physical activity predict stronger self-efficacy, both directly and indirectly through factors such as grit and resilience (Deng et al., 2023). These findings reinforce the conceptualization of physical activity as an initial driver of resource accumulation within the COR framework. Self-efficacy functions as a cognitive resource that enhances confidence, persistence, and perceived control, which together facilitate effective emotion regulation. Empirical research has shown that individuals with higher self-efficacy more frequently adopt adaptive emotion regulation strategies and report better psychological outcomes. Recent findings further suggest that emotion-regulation self-efficacy operates as a proximal mechanism in improving mental health through psychological interventions (Macrynikola et al., 2024; Int-Veen et al., 2024). These results support the theoretical assumption that self-efficacy promotes emotion regulation ability, forming a cross-type resource chain from cognition to emotion. As an emotional resource, emotion regulation directly supports mental health by shortening the duration of negative affect, facilitating cognitive reappraisal, and conserving psychological energy. Under conditions of sustained stress—such as academic overload or social isolation—strong emotion regulation consistently predicts better mental health outcomes (Menefee et al., 2022; Kökönyei et al., 2024). Among university students, the mental health benefits of physical activity are often accompanied by improved emotion regulation, indicating that emotional resources represent the most immediate link between behavioral investment and psychological well-being. Taken together, the Conservation of Resources theory predicts a serial, cross-type resource chain in which physical activity (behavioral investment) enhances self-efficacy (cognitive resource), which in turn strengthens emotion regulation (emotional resource), collectively promoting mental health. This theoretical framework supports testing the sequential pathway physical activity → self-efficacy → emotion regulation → mental health.

**Figure 1:**
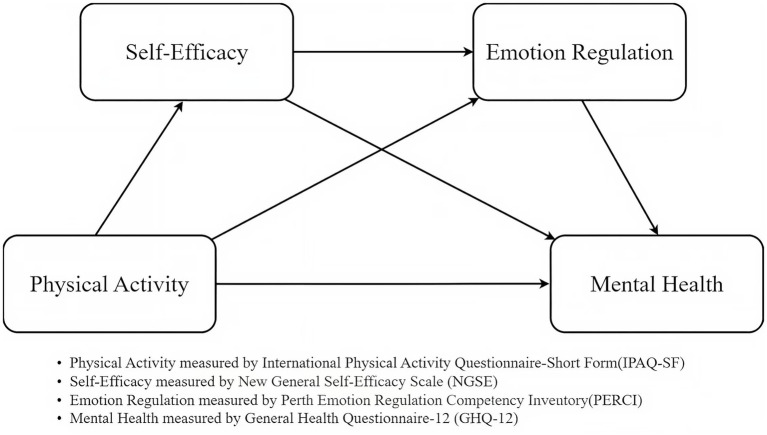
Conceptual framework.

To conclude, self-efficacy functions both as a coping belief system and a psychological scaffold for developing emotion regulation competence. The concept of a “capacity chain” facilitates understanding of complex mediation mechanisms and informs the development of targeted mental health strategies for university students. Based on the above analysis, the present study proposes a chain mediation model (as shown in Figure 1) and formulates the following hypotheses:

*H1*: Physical activity negatively predicts university students’ mental health problems.

*H2*: Self-efficacy mediates the relationship between physical activity and university students’ mental health.

*H3*: Emotion regulation ability mediates the relationship between physical activity and mental health in university students.

*H4*: Self-efficacy and emotion regulation ability play a chain mediating role in the relationship between physical activity and university students’ mental health.

For H1, based on the Conservation of Resources (COR) theory, physical activity is regarded as a proactive investment of personal resources that contributes to psychological well-being. By generating gains in physical, emotional, and cognitive resources, physical activity enhances resilience and reduces psychological distress (Hobfoll, 2010). Therefore, physical activity is hypothesized to positively predict mental health. For H2, according to social cognitive theory, repeated mastery experiences obtained through physical activity strengthen perceived competence and control, thereby enhancing self-efficacy (Hong et al., 2022). Higher self-efficacy, in turn, promotes adaptive coping and psychological stability. Thus, self-efficacy is expected to mediate the relationship between physical activity and mental health. For H3, guided by emotion regulation theory, physical activity promotes cognitive reappraisal and emotional self-control, enabling individuals to effectively manage stress and maintain affective balance (Al-Wardat et al., 2024). Accordingly, emotion regulation is hypothesized to mediate the relationship between physical activity and mental health. For H4, integrating the perspectives of COR theory and social cognitive theory, self-efficacy functions as a cognitive resource that enhances emotion regulation, which represents an emotional resource closely tied to mental health (Zhou et al., 2021; Yu et al., 2024). Hence, self-efficacy and emotion regulation are proposed to jointly mediate the relationship between physical activity and mental health in a serial pathway.

## 2 Materials and methods

### 2.1 Participants

Data were collected between March and June 2025 from three Chinese universities: Nanning Normal University, Hebei University of Chinese Medicine, and Qingdao Institute of Technology. These institutions were chosen based on research accessibility while providing reasonable diversity in geographic location (southern, northern, and eastern China) and academic orientation (teacher education, medical sciences, and engineering). This combination offered a heterogeneous student population and balanced the practical constraints of field data collection with the need for sample diversity. A cluster random sampling approach was used, treating intact academic classes as sampling units. Within each university, several classes from different year levels were randomly selected, and all students present at the time of data collection were invited to participate. Surveys were administered in person using paper-based questionnaires and collected immediately after completion to maximize response rates and ensure data quality. A total of 1,520 questionnaires were distributed, and 1,395 valid responses were retained after data cleaning, including the removal of incomplete or duplicate entries, yielding a valid response rate of 91.78%. Among the participants, 572 were male (41.0%) and 823 were female (59.0%). The average age was 19.14 years (SD = 1.23), with most respondents aged 18–20 years (86.2%), and age 18 being the most common (39.3%). By academic year, the sample included 622 freshmen (44.6%), 363 sophomores (26.0%), 281 juniors (20.1%), and 129 seniors (9.2%) (see Table 1).

**Table 1:** Demographic characteristics of participants.

Demographic variables	Category	*N*%
Gender	Male	572(41.0)
	Female	823(59.0)
Age	18	548(39.3)
	19	398(28.5)
	20	256(18.4)
	21	130(9.3)
	22	27(1.9)
	23	36(2.6)
Grade	Freshman	622(44.6)
	Sophomore	363(26.0)
	Junior	281(20.1)
	Senior	129(9.2)

The adequacy of the sample was assessed from both psychometric and statistical perspectives. According to widely accepted recommendations for factor-analytic research, the required sample size should be at least 5–10 times the number of observed items (Worthington and Whittaker, 2006). In this study, three latent-construct measures were included: the New General Self-Efficacy Scale (GSES) (8 items), the Perth Emotion Regulation Competency Inventory (32 items), and the General Health Questionnaire-12 (12 items), yielding a total of 52 items. The International Physical Activity Questionnaire–Short Form (IPAQ-SF) was treated as an index-type behavioral measure and therefore excluded from this rule. With 1,395 valid participants, the sample size substantially exceeds the recommended minimum of approximately 520, providing excellent adequacy for confirmatory factor analyses and structural equation modeling.

A *post hoc* power analysis was also conducted using G*Power (version 3.1) to verify the statistical sufficiency of the design we assumed a conservative small effect size (f^2^ = 0.02), an alpha level of 0.05, and desired power of 0.90 (Fritz and MacKinnon, 2007). Under a multiple-regression framework with three main predictors (physical activity, self-efficacy, and emotion regulation) and two control variables (gender and grade), the *a priori* required sample size was approximately 550. A sensitivity analysis further showed that, with N = 1,395, the study had 90% power to detect effects as small as f^2^ ≈ 0.007 (equivalent to a standardized *β* of about 0.08). Taken together, these results indicate that the present sample provides more than sufficient power to detect small-to-moderate direct and indirect effects within the hypothesized serial mediation model.

### 2.2 Procedure

A cross-sectional design was adopted in this study, and data collection was conducted between March and June 2025. Prior to formal questionnaire distribution, the research team communicated with relevant faculty members at the three participating universities. The study’s objectives, procedures, and confidentiality commitments were explained in detail. Upon receiving full cooperation, the team organized the on-site distribution and completion of the questionnaires. After the survey, data were exported to SPSS for cleaning and analysis; invalid responses were removed to ensure data validity and reliability. All participants were informed of the purpose and content of the study prior to participation. They voluntarily participated after signing informed consent forms in accordance with the Declaration of Helsinki. Participant anonymity was strictly maintained throughout the data collection process. The study strictly adhered to the ethical guidelines for psychological research.

### 2.3 Measures

#### 2.3.1 International physical activity questionnaire-short form

This study used the International Physical Activity Questionnaire-Short Form (IPAQ-SF) to assess the physical activity levels of university students. It is commonly utilized to measure participation time and frequency in different levels of physical activity, including vigorous, moderate, and walking, over the previous week. The total physical activity level was computed as the sum of minutes per week spent in vigorous, moderate, and walking activities. Higher total minutes reflect greater engagement in physical activity (Nascimento-Ferreira et al., 2022). The IPAQ-SF has demonstrated good reliability and validity and is widely used to assess physical activity among adolescents and adults globally. The Chinese version of the IPAQ-SF has demonstrated acceptable psychometric properties in Chinese college students when evaluated against accelerometer criteria, with moderate test–retest reliability (ICC ≈ 0.50–0.62) and fair-to-moderate concurrent validity for MVPA (*r* ≈ 0.37–0.42) (Gao et al., 2022). The IPAQ-SF is an index-type behavioral measure (minutes per week of vigorous, moderate, and walking), not a reflective latent construct. Accordingly, we did not subject it to CFA nor report internal consistency indices (*α*/*ω*), which are not meaningful for formative indices. Instead, IPAQ-SF was treated as an observed exogenous variable (total minutes/week). Its use in Chinese college samples has demonstrated acceptable test–retest reliability and fair-to-moderate criterion validity.

#### 2.3.2 New general self-efficacy scale

Self-efficacy was measured using the New General Self-Efficacy Scale (NGSE). The scale consists of 8 items rated on a 5-point Likert scale (1 = strongly disagree to 5 = strongly agree), yielding a total score ranging from 8 to 40. It evaluates an individual’s general belief in their ability to accomplish tasks and solve problems; higher scores indicate stronger self-efficacy (Chen et al., 2001). The results of the confirmatory factor analysis (CFA) indicated a good overall model fit: *χ*^2^(20) = 35.21, *p* = 0.019, CFI = 0.997, TLI = 0.996, RMSEA = 0.023, 90% CI [0.009, 0.036], SRMR = 0.012. Although the chi-square test was slightly significant, all other fit indices met or exceeded conventional cutoff criteria (CFI and TLI ≥ 0.95; RMSEA ≤ 0.05; SRMR ≤ 0.08), suggesting an excellent fit between the model and the observed data. All standardized factor loadings were statistically significant (*p* < 0.001), ranging from 0.599 to 0.700, with item-level explained variances (*R*^2^) between 0.36 and 0.49. These results indicate that the eight items reliably represent the latent construct of general self-efficacy, supporting the unidimensional factor structure of the NGSE. Based on the standardized factor loadings, the composite reliability (CR) was calculated as 0.88, and the average variance extracted (AVE) was 0.41. The CR value exceeded the recommended threshold of 0.70, demonstrating strong internal consistency. Although the AVE value was slightly below the conventional criterion of 0.50, the relatively high factor loadings and excellent model fit suggest that the convergent validity of the scale is acceptable. In addition, the internal consistency reliability, as indicated by Cronbach’s *α* = 0.88, further confirming that the NGSE exhibits high reliability and stability in this sample. Taken together, these results demonstrate that the NGSE possesses sound structural validity and reliability and can be regarded as a robust instrument for assessing general self-efficacy.

#### 2.3.3 Perth emotion regulation competency inventory

Emotion regulation ability was assessed using the Perth Emotion Regulation Competency Inventory (PERCI), which is widely used to evaluate emotional functioning among university students. The scale consists of 32 items, divided into two dimensions: regulation of negative emotions (16 items) and regulation of positive emotions (16 items), which can be combined into a general emotion regulation competency score. Each item is rated on a 5-point Likert scale (1 = not at all true, 5 = completely true). Reverse scoring is applied to specific items, and total scores are summed, with higher scores indicating stronger emotion regulation ability (Preece et al., 2018). The confirmatory factor analysis (CFA) demonstrated an excellent overall model fit: χ^2^ (463) = 789.83, *p* < 0.001, CFI = 0.980, TLI = 0.978, RMSEA = 0.016, 90% CI [0.013, 0.019], SRMR = 0.021. Although the chi-square test was significant due to the large sample size (N = 1,395), all other fit indices met or exceeded the conventional cutoff criteria (CFI and TLI ≥ 0.95; RMSEA ≤ 0.05; SRMR ≤ 0.08), indicating an excellent fit between the hypothesized two-factor model and the observed data. All standardized factor loadings were statistically significant (*p* < 0.001), with strong magnitudes across items (unstandardized loadings ranging approximately from 0.93 to 1.07 under the probit link). The items loaded clearly on their respective latent constructs—Negative Emotion Regulation (items 1–16) and Positive Emotion Regulation (items 17–32)—supporting the distinctiveness of the two correlated factors. The latent factor correlation was moderate and positive (*r* = 0.24, SE = 0.02, *p* < 0.001), suggesting that while related, the two dimensions represent conceptually distinct aspects of emotion regulation. Composite reliability (CR) for the Negative Emotion Regulation subscale was 0.91, and for the Positive Emotion Regulation subscale was 0.89, both exceeding the recommended threshold of 0.70, thus indicating strong internal consistency. The average variance extracted (AVE) values were 0.54 and 0.49, respectively. Although the AVE of the positive subscale was slightly below the conventional cutoff of 0.50, the high factor loadings and excellent overall model fit suggest that the convergent validity remains acceptable. Internal consistency reliability was further supported by Cronbach’s *α* coefficients of 0.92 (Negative) and 0.90 (Positive), demonstrating that both subscales exhibit high reliability and stability within this sample.

#### 2.3.4 General Health Questionnaire-12

Mental health status was assessed using the General Health Questionnaire-12 (GHQ-12). This scale is widely used to assess mental health levels and is suitable for psychological screening among university populations. It consists of 12 items rated on a 4-point Likert scale (0 = never, 1 = occasionally, 2 = often, 3 = almost always). Six items are positively phrased and require reverse coding. Total scores range from 0 to 36, with higher scores indicating worse mental health status (Hu et al., 2007). Although previous validation studies have suggested a cutoff of ≥14 as indicative of significant distress, in the present study the GHQ-12 score was treated as a continuous variable to represent individual differences in general mental health, consistent with common practice in non-clinical research on university students. The GHQ-12 was selected instead of longer multidimensional tools such as the SCL-90 because it is concise, psychometrically robust, and widely validated for use in large-scale university samples. The results of the confirmatory factor analysis (CFA) indicated an excellent overall model fit for the single-factor structure of the GHQ-12: *χ*^2^(54) = 191.47, *p* < 0.001, CFI = 0.987, TLI = 0.984, RMSEA = 0.037, 90% CI [0.031, 0.043], SRMR = 0.028. Although the chi-square test was statistically significant, this is common in large samples. All other fit indices met or exceeded the conventional cutoff criteria (CFI and TLI ≥ 0.95; RMSEA ≤ 0.05; SRMR ≤ 0.08), indicating a very good fit between the hypothesized model and the observed data. All standardized factor loadings were statistically significant (*p* < 0.001), ranging from 0.646 to 0.740, with item-level explained variances (*R*^2^) ranging from 0.42 to 0.55. These findings suggest that all 12 items reliably represent the latent construct of general psychological distress, providing strong support for the unidimensional factor structure of the GHQ-12. Based on the standardized factor loadings, the composite reliability (CR) was calculated as 0.93, and the average variance extracted (AVE) was 0.50. The CR value exceeded the recommended threshold of 0.70, demonstrating excellent internal consistency. The AVE reached the acceptable criterion of 0.50, indicating satisfactory convergent validity. In addition, the internal consistency reliability, as indicated by Cronbach’s *α* = 0.93, further confirmed that the GHQ-12 exhibited high reliability and stability in this sample. Taken together, these results demonstrate that the GHQ-12 possesses sound structural validity and reliability, supporting its use as a robust instrument for assessing mental health.

### 2.4 Data analysis

All data analyses were conducted using SPSS 26.0, Mplus 8.3, PROCESS 4.0, and G*Power 3.1. Preliminary data screening was performed in SPSS, including the identification and treatment of missing values and outliers, as well as variable standardization (*z*-scoring). Descriptive statistics (means, standard deviations) and bivariate correlations (Pearson’s *r*) were computed to examine the basic relationships among study variables. Spearman correlations were additionally calculated as a robustness check against potential violations of normality. Confirmatory factor analyses (CFAs) were conducted in Mplus 8.3 to assess the factorial validity of the measurement instruments. All items were treated as ordered categorical variables and estimated using the weighted least squares mean, and variance adjusted (WLSMV) method. Model fit was evaluated using multiple indices, including *χ*^2^/df, the Comparative Fit Index (CFI), Tucker–Lewis Index (TLI), Root Mean Square Error of Approximation (RMSEA) with 90% confidence intervals, and the Standardized Root Mean Square Residual (SRMR). Construct reliability and convergent validity were examined via composite reliability (CR) and average variance extracted (AVE) values. Mediation analyses were performed using Model 6 of the PROCESS macro (version 4.0; Preacher and Hayes, 2008) to test a serial mediation model in which physical activity (PA) predicted mental health (MH) through self-efficacy (SE) and emotion regulation (ER). A bias-corrected nonparametric bootstrap procedure with 5,000 resamples was used to generate 95% confidence intervals (CIs) for indirect effects. Mediation effects were considered statistically significant when the corresponding CI did not include zero. *A priori* power analysis was conducted in G*Power 3.1 based on a multiple regression framework, assuming a small effect size (*f*^2^ = 0.02), *α* = 0.05, desired power (1 − *β*) = 0.90, three predictors, and two control variables. The analysis indicated a minimum required sample size of approximately *n* = 550. The actual sample of *N* = 1,395 therefore provided adequate statistical power to detect small effects with high precision.

## 3 Results

### 3.1 Common method bias test and control

A structured questionnaire was employed to assess key variables, enhancing both the standardization and operationalization of the data. Nevertheless, self-reported data may introduce common method bias, which remains a concern in behavioral research. To minimize the influence of such bias, several procedural remedies were implemented during the study design phase. Specifically, anonymity was assured, the academic purpose of the study was emphasized, and evaluative pressure was reduced during participant recruitment and questionnaire administration. These steps were intended to mitigate participants’ inclination toward socially desirable responses. To further assess the extent to which common method bias might have affected the measurement results, Harman’s single-factor test was applied via exploratory factor analysis. All measurement items were entered into a principal component analysis without factor rotation. The analysis yielded eight components with eigenvalues greater than one. The variance explained by the first common factor was 16.495%. As these falls well below the conventional 40% cutoff, it suggests that no substantial common method bias was present in this study.

### 3.2 Descriptive statistics and correlation analysis

Correlational analysis showed that physical activity, self-efficacy, emotion regulation, and mental health were significantly associated with each other (*p* < 0.05) (as shown in Table 2).

**Table 2:** Descriptive statistics and Pearson’s correlations between the study variables.

Variable	*M*	SD	Max	Min	Physical activity	Self-efficacy	Emotion regulation	Mental health
Physical activity	1235.6961	702.1746	2495.0000	0.0000	—			
Self-efficacy	24.2789	6.6641	40.0000	8.0000	0.1069^**^	—		
Emotion regulation	90.4789	19.6999	160.0000	32.0000	0.0616^*^	0.0890^**^	—	
Mental health	14.4939	9.0454	36.0000	0.0000	−0.0828^**^	−0.1362^**^	−0.3089^**^	—

^**^Obvious correlation with precision of 0.01; ^*^Obvious correlation with precision of 0.05; M, mean; SD, standard deviation; Min, minimum; Max, maximum.

Table 2 presents the descriptive statistics and intercorrelations among the key study variables. The results indicate that physical activity, self-efficacy, emotion regulation, and mental health are significantly associated. Specifically, physical activity was positively correlated with self-efficacy (*r* = 0.1069, *p* < 0.01) and emotion regulation (*r* = 0.0616, *p* < 0.05), and negatively correlated with mental health scores (*r* = − 0.0828, *p* < 0.01), suggesting that higher levels of physical activity are linked to greater self-efficacy, stronger emotion regulation ability, and better overall mental health. All reported correlations were significant at either the 0.05 or 0.01 level.

### 3.3 Test of chain mediation effects

A chain mediation analysis was conducted with physical activity level as the independent variable, mental health level as the dependent variable, and self-efficacy and emotion regulation as sequential mediators (as shown in Table 3).

**Table 3:** Regression analysis of relationships among physical activity, self-efficacy, emotion regulation, and mental health.

Outcome variable	Predictor variable	*R*	*R* ^2^	*F*	*B*	*β*	95% CI for *β*	*t*	*p*
Self-Efficacy	Physical activity	0.1069	0.0114	16.1146	0.0010	0.1069	[0.0005, 0.0015]	4.0143	0.0001
Emotion regulation	Physical activity	0.1032	0.0107	7.4977	0.0015	0.0526	[0.0001, 0.0030]	1.9634	0.0498
	Self-efficacy	—	—	—	0.2464	0.0834	[0.0909, 0.4019]	3.1087	0.0019
Mental health (total effect)	Physical activity	0.0828	0.0069	9.62	−0.0011	−0.0828	[−0.0017, −0.0004]	−3.1016	0.002
Mental health (direct effect)	Physical activity	0.3319	0.1102	57.4111	−0.0007	−0.0534	[−0.0013, −0.0001]	−2.0977	0.0361
	Self-efficacy	—	—	—	−0.1414	−0.1042	[−0.2093, −0.0734]	−4.0801	<0.0001
	Emotion regulation	—	—	—	−0.1361	−0.2964	[−0.1590, −0.1132]	−11.6556	<0.0001

B = Unstandardized regression coefficient; β = Standardized regression coefficient; CI = Confidence Interval; R = Multiple correlation coefficient; R^2^ = Coefficient of determination; F = *F*-test statistic; t = *t*-test statistic; p = Probability value.

**Figure 2:**
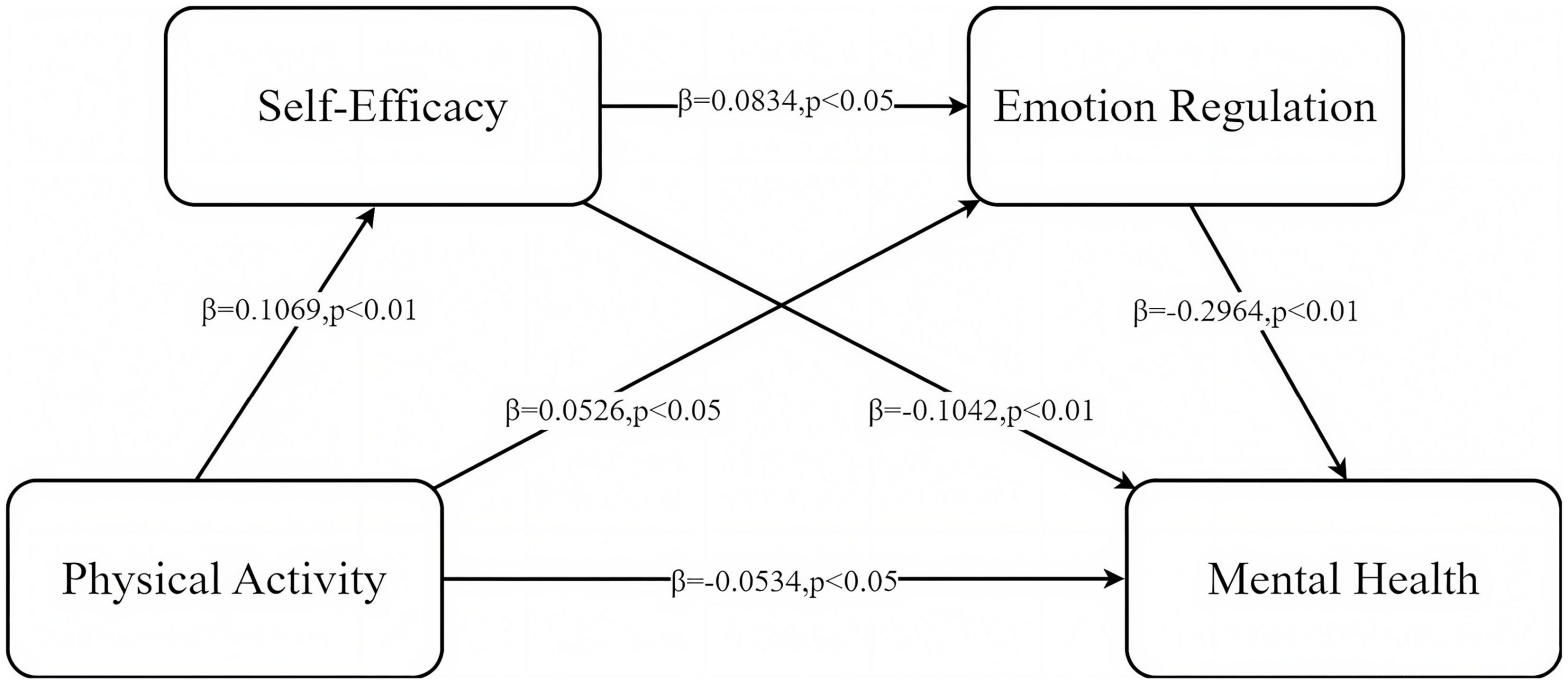
The chained mediation model of the self-efficacy and emotion regulation in physical activity and mental health.

According to the regression results presented in Table 3, physical activity, self-efficacy, and emotion regulation all exhibited statistically significant effects in predicting university students’ mental health. Physical activity significantly and negatively predicted mental health (*B* = −0.0011, *β* = −0.0828, *t* = −3.1016, *p* = 0.0020), indicating that higher levels of physical activity were associated with lower levels of psychological distress. In the mediation pathway, physical activity significantly predicted self-efficacy (*B* = 0.0010, *β* = 0.1069, *t* = 4.0143, *p* = 0.0001), suggesting that students who engage in regular physical activity have greater confidence and a stronger sense of control when facing challenges. Furthermore, self-efficacy positively predicted emotion regulation (*B* = 0.2464, *β* = 0.0834, *t* = 3.1087, *p* = 0.0019), indicating that individuals with higher self-efficacy are more adept at employing adaptive strategies to regulate their emotions. Although the effect of physical activity on emotion regulation approached significance (*B* = 0.0015, *β* = 0.0526, *t* = 1.9634, *p* = 0.0498), this finding still supports the potential direct promotive influence of physical activity on emotional regulation capacity. This dual-pathway mechanism reinforces the role of physical activity in enhancing both self-efficacy and emotion regulation among university students. In the final multiple regression model predicting mental health, physical activity (*B* = −0.0007, *β* = −0.0534, *t* = −2.0977, *p* = 0.0361), self-efficacy (*B* = −0.1414, *β* = −0.1042, *t* = −4.0801, *p* < 0.0001), and emotion regulation (*B* = −0.1361, *β* = −0.2964, *t* = −11.6556, *p* < 0.0001) all significantly predicted mental health. The overall model demonstrated a good fit (*R*^2^ = 0.1102, *F* = 57.4111, *p* < 0.0001). Notably, emotion regulation had the largest standardized regression coefficient (*β* = −0.2964), indicating its central role in explaining variations in mental health. The specific chain mediation pathway through which self-efficacy and emotion regulation mediate the relationship between physical activity and mental health among university students is shown below (as shown in Figure 2).

A bias-corrected bootstrap approach was applied to evaluate the robustness of the mediation effects. The analysis revealed that the total effect of physical activity on mental health was −0.0808, with the overall indirect effects accounting for 35.51% of the total effect. Specifically, the indirect effect via self-efficacy (PA → SE → MH) was −0.0111, explaining 13.28% of the total effect, whereas the indirect effect via emotion regulation (PA → ER → MH) was −0.0156, explaining 18.66%. The sequential mediation pathway (PA → SE → ER → MH) yielded an indirect effect of −0.0026, representing 3.57% of the total effect. All three 95% bias-corrected bootstrap confidence intervals excluded zero, indicating that the indirect effects were statistically significant. The direct effect (−0.0534) remained significant and accounted for 64.49% of the total effect. These findings demonstrate that self-efficacy and emotion regulation serve both independent and sequential mediating roles in the association between physical activity and mental health (see Table 4).

**Table 4:** Mediation analysis of the effects of physical activity on mental health through self-efficacy and emotion regulation.

Effect type	Effect (standardized)	SE	95% confidence interval	Relative proportion
Total indirect effect	−0.0294	0.0088	[−0.0471, −0.0124]	35.51%
Ind1 (PA → SE → MH)	−0.0111	0.0041	[−0.0199, −0.0041]	13.28%
Ind2 (PA → ER → MH)	−0.0156	0.0078	[−0.0314, −0.0005]	18.66%
Ind3 (PA → SE → ER → MH)	−0.0026	0.0012	[−0.0052, −0.0008]	3.57%
Direct effect	−0.0534	0.0254	[−0.1032, −0.0036]	64.49%
Total effect	−0.0828	0.0267	[−0.1351, −0.0305]	100%

PA, physical activity; SE, self-efficacy; ER, emotion regulation; MH, mental health; BootSE, bootstrapped standard error; BootLLCI/BootULCI, 95% bootstrapped confidence interval limits; Proportion, percentage of the total effect mediated. The PROCESS output did not provide the standard errors (SE) for the standardized direct and total effects; therefore, they are denoted with an em dash (—).

To further evaluate the magnitude of the mediation effects, Cohen’s *f*^2^ was calculated for each indirect pathway. The indirect effect through self-efficacy (PA → SE → MH) showed a small effect size (*f*^2^ = 0.04), while the indirect effect through emotion regulation (PA → ER → MH) indicated a medium effect size (*f*^2^ = 0.08). The sequential mediation pathway (PA → SE → ER → MH) exhibited a small effect size (*f*^2^ = 0.03). According to research (Selya et al., 2012), *f*^2^ values of 0.02, 0.15, and 0.35 represent small, medium, and large effects, respectively. These results suggest that, although the indirect effects are statistically significant, their magnitudes are in the small-to-medium range, implying that physical activity influences mental health partly but not overwhelmingly through these psychological mechanisms.

## 4 Discussion

This study investigated the mechanisms linking physical activity and mental health among college students by examining the mediating roles of self-efficacy and emotion regulation. The results showed that physical activity had both direct and indirect effects on mental health (*β* = −0.0828, *p* < 0.01), with indirect effects through self-efficacy (13.28%), emotion regulation (18.67%), and their sequential combination (3.57%). These findings partially supported the proposed hypotheses (H1–H5) and provided empirical evidence for a cross-resource mechanism consistent with Conservation of Resources (COR) theory.

### 4.1 Direct relationship between physical activity and university students’ mental health

Physical activity showed a significant negative association with mental health problems (*β* = −0.0828, *p* < 0.01), indicating that higher levels of physical activity were related to better psychological well-being. The findings demonstrate that physical activity serves as a significant predictor of enhanced mental health in university students, particularly in mitigating negative psychological states. Extensive empirical research has consistently revealed an inverse relationship between regular exercise and mental health problems, whereby higher physical activity levels correlate with reduced symptoms of depression, anxiety, and stress. Engaging in 150–300 min of moderate-intensity physical activity per week is sufficient to produce significant mood-related improvements. Conversely, over-exercising or compulsive physical activity may result in exercise addiction, mental fatigue, and a rebound in anxiety symptoms (White et al., 2024; Wassenaar et al., 2021; Rahmati et al., 2024). These findings have been replicated across various cultural and educational settings, indicating the context-independent protective effect of physical activity on mental health. Moderate aerobic or resistance training can upregulate brain-derived neurotrophic factor (BDNF) and neurotransmitters such as dopamine and serotonin while reducing cortisol levels. These changes contribute to greater emotional stability and faster recovery from stress (Kandola et al., 2019). Neuroimaging studies further indicate that long-term exercise increases prefrontal cortex thickness and improves limbic system functioning. These structural and functional changes are closely associated with better emotional control and cognitive regulation skills (Wu et al., 2022). Studies suggest that the sense of accomplishment and control achieved through exercise contributes to increased self-esteem and self-efficacy. Participation in team or community-based sports expands social bonds and offers avenues for emotional support. Additionally, the reciprocal relationship between exercise and sleep quality plays a buffering role in emotional instability by enhancing restfulness (Atoui et al., 2021). In this sense, physical activity not only stimulates physiological responses but also promotes positive emotional experiences and a sense of belonging, offering dual reinforcement for mental well-being. In conclusion, physical activity serves as a cost-effective, multidimensional, and non-pharmacological approach to psychological well-being enhancement.

### 4.2 The mediating role of self-efficacy between physical activity and mental health

The mediation analysis revealed that self-efficacy significantly mediated the relationship between physical activity and mental health (indirect effect = −0.0111, 13.28% of total effect, 95% CI [−0.0199, −0.0041]). The present findings underscore the pivotal role of self-efficacy as a mediator linking physical activity to mental health in university students, contributing novel evidence. This aligns with the Conservation of Resources theory, which posits that physical activity, as an external behavioral investment, activates and accumulates self-efficacy (a core cognitive resource), thereby sustaining mental health through a “gain spiral” (Hobfoll, 1989). Physical activity significantly enhances self-efficacy, which in turn buffers stress, reduces symptoms of depression and anxiety, and improves subjective well-being (Tikac et al., 2022). According to Social Cognitive Theory, self-efficacy is formed through a positive feedback cycle involving mastery experiences, self-evaluation, and the reconstruction of efficacy beliefs (Locke, 1997). Even short-term interventions (4–6 weeks) can significantly improve general or exercise-specific self-efficacy, and as interventions persist, these benefits may transfer to non-physical domains such as learning and social interaction (Mu et al., 2024). Thus, optimizing mental health outcomes through exercise requires intentionally embedding self-efficacy enhancement strategies within physical activity programs. Such an approach offers actionable guidance for the design of effective mental health interventions in university contexts. The correlation between physical activity and self-efficacy observed in this study (*r* = 0.1069, *p* < 0.01) was lower than that reported in previous research (approximately *r* ≈ 0.41) (Zhang et al., 2024). This discrepancy may arise from differences in how physical activity was operationalized. The present study employed the International Physical Activity Questionnaire–Short Form (IPAQ-SF), which captures a wide range of everyday activities such as walking, commuting, and household chores. In contrast, earlier studies often focused on structured exercise interventions, including supervised aerobic or resistance training programs, that provide stronger mastery experiences and clearer performance feedback. Consequently, the lower correlation found here may reflect the diffuse, less goal-oriented nature of daily activity, which may foster weaker self-efficacy enhancement compared with structured exercise contexts.

### 4.3 The mediating role of emotion regulation in the relationship between physical activity and mental health

Emotion regulation also exerted a significant mediating effect between physical activity and mental health (indirect effect = −0.0156, 18.66% of total effect, 95% CI [−0.0314, −0.0005]). The present study highlights the key mediating function of emotion regulation in the physical activity–mental health relationship, offering novel empirical support. This finding is consistent with prior research indicating that emotion regulation mediates the relationship between physical activity and mental health in children and adolescents (Sheng et al., 2024; Tang et al., 2022; Zhao et al., 2025). Emotion regulation ability directly influences how individuals process and sustain negative emotions and is considered a transdiagnostic risk factor for both depression and anxiety. Higher levels of emotion regulation ability help shorten the duration of negative affect, suppress rumination and stress consumption, sustain positive emotion, and promote social adaptation. Moreover, it allows individuals to seek and obtain social support through appropriate emotional communication (Ramos-Cejudo et al., 2024). Thus, when physical activity first enhances emotion regulation, it subsequently promotes mental health across emotional, cognitive, and social domains. Previous studies have largely focused on the direct link between physical activity and emotional states or have treated emotion regulation difficulties as outcome variables. In contrast, the current study treats emotion regulation as an active, malleable capacity, emphasizing its plasticity and mediating function. This is consistent with Wu et al.’s findings in early adult samples and extends the applicability of these results to Chinese university students (Wu et al., 2022). Mind–body interventions such as yoga and tai chi show strong potential for enhancing emotional acceptance and awareness. Conversely, high-intensity interval training may be more effective in strengthening emotional restraint and impulse regulation. University physical education programs may integrate emotion recognition and regulation training. Combining physical activation with emotional introspection may optimize the synergistic effects of exercise and emotion regulation.

Within the framework of the Conservation of Resources (COR) theory, the stronger mediating role of emotion regulation may be explained by the immediacy and universality of emotional resources in preserving psychological equilibrium. Emotional resources—such as adaptive regulation strategies, emotional clarity, and acceptance—operate at the front line of stress response and therefore exert a more direct protective influence on mental health than cognitive resources like self-efficacy. While self-efficacy facilitates resource gain by shaping motivation and behavioral persistence, emotion regulation determines how efficiently individuals conserve existing resources under emotional strain (Hobfoll et al., 2018). In this sense, emotion regulation functions as a “first-order” defense mechanism that buffers stress before cognitive resources are mobilized. Regular physical activity may enhance this emotional buffer by promoting neurophysiological stability and reinforcing adaptive affective patterns (De Nys et al., 2022). Consequently, improvements in emotion regulation capacity translate more rapidly and strongly into mental health benefits than enhancements in self-efficacy, explaining why its mediating effect appears most pronounced in the present study.

### 4.4 The chain mediating role of self-efficacy and emotion regulation ability

The sequential mediation pathway “Physical Activity → Self-Efficacy → Emotion Regulation → Mental Health” was statistically significant but relatively small (indirect effect = −0.0026, 3.57% of total effect, 95% CI [−0.0052, −0.0008]). This study is the first to empirically validate the chain mediation model “Physical Activity → Self-Efficacy → Emotion Regulation → Mental Health” in a university student sample. The findings illuminate how self-efficacy and emotion regulation develop in a staged, synergistic manner within physical activity settings. The sense of mastery generated through physical activity first enhances self-efficacy. Elevated self-efficacy enhances individuals’ awareness of emotional signals and their capacity for strategic emotional management. These cascading effects help alleviate depressive and anxious symptoms, thereby promoting better mental health outcomes. Exercise environments provide immediate positive feedback and measurable progress, which strengthen belief in one’s capabilities (Locke, 1997). Positive somatic and psychological experiences induced by exercise contribute to enhanced self-efficacy (Mu et al., 2024). Once individuals believe “I can do it,” they are more motivated to apply effortful emotional regulation strategies. Individuals with high self-efficacy display greater emotional awareness and strategy diversity in emotionally charged situations. Such individuals prefer adaptive strategies like reappraisal and acceptance over maladaptive ones like suppression or avoidance (Ramos-Cejudo et al., 2024). Self-efficacy may not be an independent parallel resource but rather a catalyst in the transformation of emotion regulation capacity. By enhancing regulatory control, self-efficacy enables individuals to suppress or resolve negative emotions when they arise. Emotion regulation ability directly influences the duration of negative affect and the depth of rumination. This ability is a well-established transdiagnostic predictor of internalizing disorders such as anxiety and depression. When self-efficacy activates emotion regulation, individuals become more effective in using strategies like reappraisal or attentional diversion during peak stress. This prevents the emotional overconsumption that contributes to mental fatigue or burnout. Within the framework of COR theory, this chain pathway illustrates a closed loop of “resource acquisition–transformation–amplification.” External behavioral investment (physical activity) activates cognitive resources (self-efficacy), which in turn facilitates the development of emotional resources (emotion regulation ability), and the synergy between these resources leads to psychological homeostasis. Previous models often relied on single-path explanations, overlooking the dual-channel mediating potential of self-efficacy and emotion regulation. The current findings bridge this gap and provide empirical support for a comprehensive psychological mechanism of exercise benefits. Beyond its statistical significance, the sequential mediation model yields important theoretical insights. It suggests that self-efficacy and emotion regulation are not parallel or independent mechanisms but dynamically intertwined processes through which physical activity influences mental health. Within the framework of the Conservation of Resources (COR) theory, this finding reflects a cross-domain resource transmission, in which behavioral engagement in physical activity cultivates cognitive resources such as self-efficacy, which subsequently facilitate the development of emotional resources like regulation capacity. This sequential conversion represents a cascade of resource activation, transformation, and reinforcement, highlighting that the psychological benefits of exercise extend beyond transient mood improvements to more enduring adaptive functioning.

Moreover, the relatively small proportion of the sequential mediation effect (3.14%) suggests that the interaction between self-efficacy and emotion regulation in transmitting the benefits of physical activity to mental health is relatively modest. This attenuated effect could stem from unmodeled moderating variables, such as social support, personality traits, or motivational climate. For instance, individuals with strong social support may directly regulate their emotions through interpersonal reassurance rather than relying on self-efficacy-based strategies, thereby diminishing the strength of the PA → SE → ER → MH chain pathway. Future research should consider testing moderated mediation models to clarify how contextual factors influence the strength and direction of this sequential mechanism.

### 4.5 Theoretical implications

The findings of this study extend the Conservation of Resources (COR) theory by empirically validating a cross-domain resource chain linking behavioral, cognitive, and emotional resources. Traditional applications of COR theory have primarily emphasized resource accumulation within a single domain—such as psychological or social resources. In contrast, the present results demonstrate that behavioral engagement in physical activity can trigger cognitive gains (self-efficacy), which in turn facilitate emotional resource enhancement (emotion regulation). The relative strength of the emotion regulation pathway underscores the hierarchical organization of resources in the COR framework: emotional resources function as immediate protective assets that buffer stress, whereas cognitive resources like self-efficacy serve as generative catalysts for long-term resilience. By mapping these inter-domain resource conversions, this study enriches COR theory with a more integrative perspective that captures how behavioral, cognitive, and affective systems jointly sustain psychological well-being.

## 5 Limitations and future directions

This study employed a cross-sectional design, which precludes any inference of causal relationships among physical activity, self-efficacy, emotion regulation, and mental health. The observed associations should therefore be interpreted as correlational rather than causal. To establish temporal ordering and test potential causal mechanisms, future research should employ longitudinal or experimental designs. All core variables were measured through self-report questionnaires, which may introduce common method variance, social desirability bias, and recall bias. Future studies could benefit from incorporating multi-method assessments, such as behavioral measures, ecological momentary assessment (EMA), or physiological indicators, to enhance data validity. Although the sample was drawn from multiple universities, it consisted exclusively of currently enrolled undergraduate students, without differentiation by academic major, physical education background, or athletic experience. This sampling constraint may limit the generalizability of the findings to broader populations, including postgraduate students or non-student groups. Future research should aim to include more diverse samples across educational, occupational, and cultural contexts to verify the robustness and applicability of the present findings.

## 6 Conclusion

The findings validate a sequential mediation pathway in which physical activity influences mental health through self-efficacy and emotion regulation among university students. Physical activity, recognized as a key positive health behavior, demonstrates not only direct benefits for mental health but also indirect effects by mobilizing internal psychological assets. Based on a sequential mediation model, this study further clarified that self-efficacy and emotion regulation function as separate mediators and may collectively form a sequential mediating chain between physical activity and mental health. This finding not only extends the theoretical dimension of how physical activity operates but also enriches the application of Conservation of Resources theory in exercise psychology by revealing the internal logic of mental health enhancement through capacity accumulation.

From a practical perspective, the results provide clear direction for developing university-based mental health promotion programs by integrating physical training with psychological skill development to systematically enhance students’ psychological adaptability and overall well-being. Given the pronounced mediating role of emotion regulation, universities could implement integrated mind–body interventions that deliberately pair physiological activation with emotional skill training. A feasible format might include 30 min of moderate-intensity aerobic activity immediately followed by 10 min of guided emotional awareness and cognitive reappraisal practice. Such programs foster neurophysiological balance while simultaneously strengthening adaptive emotional responses, thereby amplifying the psychological returns of physical activity. Secondly, recognizing the mediating function of self-efficacy, progressive mastery-oriented exercise regimens can be employed to sustain motivation and perceived competence. For example, students might begin with three weekly 20-min walking sessions and gradually progress to three 30-min jogging sessions. Incremental goal setting, feedback loops, and self-monitoring tools can further consolidate efficacy beliefs and promote adherence.

## Data Availability

The original contributions presented in the study are included in the article/supplementary material, further inquiries can be directed to the corresponding author.

## References

[ref1] Albert B. (2012). On the functional properties of perceived self-efficacy revisited. J. Manage. 38, 9–44. doi: 10.1177/0149206311410606

[ref2] Al-Wardat M. Salimei C. Alrabbaie H. Etoom M. Khashroom M. Clarke C. . (2024). Exploring the links between physical activity, emotional regulation, and mental well-being in Jordanian university students. J. Clin. Med. 13:1533. doi: 10.3390/jcm13061533, PMID: 38541759 PMC10970980

[ref3] Atoui S. Chevance G. Romain A. J. Kingsbury C. Lachance J. P. Bernard P. (2021). Daily associations between sleep and physical activity: a systematic review and meta-analysis. Sleep Med. Rev. 57:101426. doi: 10.1016/j.smrv.2021.101426 33571893

[ref4] Bardeen J. R. Fergus T. A. (2020). Emotion regulation self-efficacy mediates the relation between happiness emotion goals and depressive symptoms: a cross-lagged panel design. Emotion 20, 910–915. doi: 10.1037/emo0000592, PMID: 30816743

[ref5] Chen G. Gully S. M. Eden D. (2001). Validation of a new general self-efficacy scale. Organ. Res. Methods 4, 62–83. doi: 10.1177/109442810141004

[ref6] De Nys L. Anderson K. Ofosu E. F. Ryde G. C. Connelly J. Whittaker A. C. (2022). The effects of physical activity on cortisol and sleep: a systematic review and meta-analysis. Psychoneuroendocrinology 143:105843. doi: 10.1016/j.psyneuen.2022.105843, PMID: 35777076

[ref7] Deng J. Liu Y. Chen R. Wang Y. (2023). The relationship between physical activity and life satisfaction among university students in China: the mediating role of self-efficacy and resilience. Behav. Sci. 13:889. doi: 10.3390/bs13110889, PMID: 37998636 PMC10669265

[ref8] Di Giunta L. Lunetti C. Gliozzo G. Rothenberg W. A. Lansford J. E. Eisenberg N. . (2022). Negative parenting, adolescents’ emotion regulation, self-efficacy in emotion regulation, and psychological adjustment. Int. J. Environ. Res. Public Health 19:2251. doi: 10.3390/ijerph19042251, PMID: 35206436 PMC8871997

[ref9] Egozi Farkash H. Lahad M. Hobfoll S. E. Leykin D. Aharonson-Daniel L. (2022). Conservation of resources, psychological distress, and resilience during the COVID-19 pandemic. Int. J. Public Health 67:1604567. doi: 10.3389/ijph.2022.1604567, PMID: 36119444 PMC9472268

[ref10] Fritz M. S. MacKinnon D. P. (2007). Required sample size to detect the mediated effect. Psychol. Sci. 18, 233–239. doi: 10.1111/j.1467-9280.2007.01882.x, PMID: 17444920 PMC2843527

[ref11] Gao H. Li X. Zi Y. Mu X. Fu M. Mo T. . (2022). Reliability and validity of common subjective instruments in assessing physical activity and sedentary behaviour in Chinese college students. Int. J. Environ. Res. Public Health 19:8379. doi: 10.3390/ijerph19148379, PMID: 35886229 PMC9320576

[ref12] Gross J. J. (1998). The emerging field of emotion regulation: an integrative review. Rev. Gen. Psychol. 2, 271–299. doi: 10.1037/1089-2680.2.3.271

[ref13] Hobfoll S. E. (1989). Conservation of resources. A new attempt at conceptualizing stress. Am. Psychol. 44, 513–524. doi: 10.1037//0003-066x.44.3.513, PMID: 2648906

[ref14] Hobfoll S. E. (2001). The influence of culture, community, and the nested-self in the stress process: advancing conservation of resources theory. Appl. Psychol. 50, 337–421. doi: 10.1111/1464-0597.00062

[ref15] Hobfoll S. E. (2010). Conservation of resources theory: Its implication for stress, health, and resilience. New York, NY: Oxford University Press, 486.

[ref16] Hobfoll S. E. (2011). Conservation of resource caravans and engaged settings. J. Occup. Organ. Psychol. 84, 116–122. doi: 10.1111/j.2044-8325.2010.02016.x

[ref17] Hobfoll S. E. Halbesleben J. Neveu J. P. Westman M. (2018). Conservation of resources in the organizational context: the reality of resources and their consequences. Annu. Rev. Organ. Psychol. Organ. Behav. 5, 103–128. doi: 10.1146/annurev-orgpsych-032117-104640

[ref18] Hong J. Mreydem H. W. Abou Ali B. T. Saleh N. O. Hammoudi S. F. Lee J. . (2022). Mediation effect of self-efficacy and resilience on the psychological well-being of Lebanese people during the crises of the COVID-19 pandemic and the Beirut explosion. Front. Psych. 12:3578. doi: 10.3389/fpsyt.2021.733578, PMID: 35082699 PMC8784513

[ref19] Hu Y. Stewart-Brown S. Twigg L. Weich S. (2007). Can the 12-item general health questionnaire be used to measure positive mental health? Psychol. Med. 37, 1005–1013. doi: 10.1017/s0033291707009993, PMID: 17274855

[ref20] Int-Veen I. Volz M. Kroczek A. Fallgatter A. J. Ehlis A. C. Rubel J. A. . (2024). Emotion regulation use in daily-life and its association with success of emotion-regulation, self-efficacy, stress, and state rumination. Front. Psychol. 15:15. doi: 10.3389/fpsyg.2024.1400223, PMID: 39502151 PMC11534797

[ref21] Kandola A. Ashdown-Franks G. Hendrikse J. Sabiston C. M. Stubbs B. (2019). Physical activity and depression: towards understanding the antidepressant mechanisms of physical activity. Neurosci. Biobehav. Rev. 107, 525–539. doi: 10.1016/j.neubiorev.2019.09.040, PMID: 31586447

[ref22] Kökönyei G. Kovács L. N. Szabó J. Urbán R. (2024). Emotion regulation predicts depressive symptoms in adolescents: a prospective study. J Youth Adolesc. 53, 142–158. doi: 10.1007/s10964-023-01894-4, PMID: 37985558 PMC10761508

[ref23] Krifa I. van Zyl L. E. Braham A. Ben Nasr S. Shankland R. (2022). Mental health during COVID-19 pandemic: the role of optimism and emotional regulation. Int. J. Environ. Res. Public Health 19:1413. doi: 10.3390/ijerph19031413, PMID: 35162435 PMC8835172

[ref24] Kristensen S. M. Larsen T. M. B. Urke H. B. Danielsen A. G. (2023). Academic stress, academic self-efficacy, and psychological distress: a moderated mediation of within-person effects. J Youth Adoles 52, 1512–1529. doi: 10.1007/s10964-023-01770-1, PMID: 36995523 PMC10175374

[ref25] Lande N. M. Ask T. F. Sætren S. S. Lugo R. G. Sütterlin S. (2023). The role of emotion regulation for general self-efficacy in adolescents assessed through both neurophysiological and self-reported measures. Psychol. Res. Behav. Manag. 16, 3373–3383. doi: 10.31234/osf.io/abxfn 37650113 PMC10464900

[ref26] Li W. Quan S. (2025). Mediating effects of resilience on regulatory emotional self-efficacy and adverse mental health outcomes among college students in China. Sci. Rep. 15:25168. doi: 10.1038/s41598-025-09260-z, PMID: 40646071 PMC12254346

[ref27] Li Y. Wang A. Wu Y. Han N. Huang H. (2021). Impact of the COVID-19 pandemic on the mental health of college students: a systematic review and Meta-analysis. Front. Psychol. 12. doi: 10.3389/fpsyg.2021.669119, PMID: 34335381 PMC8316976

[ref28] Li X. Wang J. Yu H. Liu Y. Xu X. Lin J. . (2024). How does physical activity improve adolescent resilience? Serial indirect effects via self-efficacy and basic psychological needs. PeerJ. 12:e17059. doi: 10.7717/peerj.17059, PMID: 38436018 PMC10909365

[ref29] Locke E. A. (1997). Self-efficacy: the exercise of control. Person. Psychol. 50, 801–804.

[ref30] Macrynikola N. Chang S. Torous J. (2024). Emotion regulation self-efficacy as a mechanism of alliance and outcomes in a brief, transdiagnostic digital mental health intervention: L’auto-efficacité de la régulation des émotions en tant que mécanisme d’alliance et de résultats dans une brève intervention transdiagnostique numérique en santé mentale. Can. J. Psychiatr. 70, 824–833. doi: 10.1177/07067437241274201, PMID: 39308411 PMC11562972

[ref31] Menefee D. S. Ledoux T. Johnston C. A. (2022). The importance of emotional regulation in mental health. Am. J. Lifestyle Med. 16, 28–31. doi: 10.1177/15598276211049771, PMID: 35185423 PMC8848120

[ref32] Mikkelsen K. Stojanovska L. Polenakovic M. Bosevski M. Apostolopoulos V. (2017). Exercise and mental health. Maturitas 106, 48–56. doi: 10.1016/j.maturitas.2017.09.003, PMID: 29150166

[ref33] Mu F.-Z. Liu J. Lou H. Zhu W.-D. Wang Z.-C. Li B. (2024). How breaking a sweat affects mood: the mediating role of self-efficacy between physical exercise and emotion regulation ability. PLoS One 19:e0303694. doi: 10.1371/journal.pone.0303694 38870188 PMC11175485

[ref34] Nascimento-Ferreira M. V. Rosa A. C. A. Azevedo J. C. Santos A. R. d. A. De Araujo-Moura K. Ferreira K. A. (2022). Psychometric properties of the online international physical activity questionnaire in college students. Int. J. Environ. Res. Public Health 19:15380. doi: 10.3390/ijerph192215380 36430099 PMC9690787

[ref9001] Preacher K. J. Hayes A. F. (2008). Asymptotic and resampling strategies for assessing and comparing indirect effects in multiple mediator models. Behav. Res. Methods 40, 879–891. doi: 10.3758/BRM.40.3.879 18697684

[ref35] Preece D. A. Becerra R. Robinson K. Dandy J. Allan A. (2018). Measuring emotion regulation ability across negative and positive emotions: the Perth emotion regulation competency inventory (PERCI). Pers. Individ. Differ. 135, 229–241. doi: 10.1016/j.paid.2018.07.025

[ref36] Rahmati M. Lee S. Yon D. K. Lee S. W. Udeh R. McEvoy M. . (2024). Physical activity and prevention of mental health complications: an umbrella review. Neurosci. Biobehav. Rev. 160:105641. doi: 10.1016/j.neubiorev.2024.105641, PMID: 38527637

[ref37] Ramos-Cejudo J. Salguero J. M. García-Sancho E. Gross J. J. (2024). Emotion regulation frequency and self-efficacy: differential associations with affective symptoms. Behav. Ther. 55, 1004–1014. doi: 10.1016/j.beth.2024.02.009, PMID: 39174261

[ref38] Ryan R. M. Soenens B. Vansteenkiste M. (2019). Reflections on self-determination theory as an organizing framework for personality psychology: interfaces, integrations, issues, and unfinished business. J. Pers. 87, 115–145. doi: 10.1111/jopy.12440, PMID: 30325499

[ref39] Sadeghi Bahmani D. Razazian N. Motl R. W. Farnia V. Alikhani M. Pühse U. . (2020). Physical activity interventions can improve emotion regulation and dimensions of empathy in persons with multiple sclerosis: an exploratory study. Mult. Scler. Relat. Disord. 37:101380. doi: 10.1016/j.msard.2019.101380, PMID: 32173007

[ref40] Selya A. S. Rose J. S. Dierker L. C. Hedeker D. Mermelstein R. J. (2012). A practical guide to calculating Cohen’s f2, a measure of local effect size, from PROC MIXED. Front. Psychol. 3:3. doi: 10.3389/fpsyg.2012.00111, PMID: 22529829 PMC3328081

[ref41] Sheng X. Wen X. Liu J. Zhou X. Li K. (2024). Effects of physical activity on anxiety levels in college students: mediating role of emotion regulation. PeerJ. 12:e17961. doi: 10.7717/peerj.17961, PMID: 39308821 PMC11416097

[ref42] Sun X. Wang Z. J. Li Y. Y. Chan K. Q. Miao X. Y. Zhao S. . (2022). Trends of college students’ mental health from 2005 to 2019 and its rural-urban disparities in China. J. Affect. Disord. 302, 160–169. doi: 10.1016/j.jad.2022.01.042, PMID: 35033592

[ref43] Tang S. Chen H. Wang L. Lu T. Yan J. (2022). The relationship between physical exercise and negative emotions in college students in the post-epidemic era: the mediating role of emotion regulation self-efficacy. Int. J. Environ. Res. Public Health 19:12166. doi: 10.3390/ijerph191912166, PMID: 36231469 PMC9566100

[ref44] Tikac G. Unal A. Altug F. (2022). Regular exercise improves the levels of self-efficacy, self-esteem and body awareness of young adults. J. Sports Med. Phys. Fitness 62, 157–161. doi: 10.23736/s0022-4707.21.12143-7, PMID: 33555673

[ref45] Tse A. C. Y. (2020). Brief report: impact of a physical exercise intervention on emotion regulation and behavioral functioning in children with autism spectrum disorder. J. Autism Dev. Disord. 50, 4191–4198. doi: 10.1007/s10803-020-04418-2 32130593

[ref46] Wassenaar T. M. Wheatley C. M. Beale N. Nichols T. Salvan P. Meaney A. . (2021). The effect of a one-year vigorous physical activity intervention on fitness, cognitive performance and mental health in young adolescents: the fit to study cluster randomised controlled trial. Int. J. Behav. Nutr. Phys. Act. 18:47. doi: 10.1186/s12966-021-01113-y, PMID: 33789683 PMC8011147

[ref47] White R. L. Vella S. Biddle S. Sutcliffe J. Guagliano J. M. Uddin R. . (2024). Physical activity and mental health: a systematic review and best-evidence synthesis of mediation and moderation studies. Int. J. Behav. Nutr. Phys. Act. 21:134. doi: 10.1186/s12966-024-01676-6, PMID: 39609855 PMC11603721

[ref48] Worthington R. L. Whittaker T. A. (2006). Scale development research: a content analysis and recommendations for best practices. Counsel. Psychol. 34, 806–838. doi: 10.1177/0011000006288127

[ref49] Wu J. Zhu L. Dong X. Sun Z. Cai K. Shi Y. . (2022). Relationship between physical activity and emotional regulation strategies in early adulthood: mediating effects of cortical thickness. Brain Sci. 12:1210. doi: 10.3390/brainsci12091210, PMID: 36138946 PMC9496840

[ref50] Xu J. Xu Y. (2025). The impact of self-efficacy on psychological resilience in EFL learners: a serial mediation model. BMC Psychol. 13:858. doi: 10.1186/s40359-025-03236-4, PMID: 40754539 PMC12320316

[ref51] Yang Z. Y. Wang Y. T. Xia L. Zheng Y. C. Feng Z. Z. (2022). The relationships between prospection, self-efficacy, and depression in college students with cross-lagged analysis. Int. J. Environ. Res. Public Health 19:14685. doi: 10.3390/ijerph192214685, PMID: 36429404 PMC9690034

[ref52] Yu H. Zhu T. Tian J. Zhang G. Wang P. Chen J. . (2024). Physical activity and self-efficacy in college students: the mediating role of grit and the moderating role of gender. PeerJ. 12:e17422. doi: 10.7717/peerj.17422, PMID: 38803579 PMC11129692

[ref53] Zhang G. Feng W. Zhao L. Zhao X. Li T. (2024). The association between physical activity, self-efficacy, stress self-management and mental health among adolescents. Sci. Rep. 14:5488. doi: 10.1038/s41598-024-56149-4, PMID: 38448518 PMC10917799

[ref54] Zhao G. Xiao L. r. Chen Y. h. Zhang M. Peng K. w. Wu H. m. (2025). Association between physical activity and mental health problems among children and adolescents: a moderated mediation model of emotion regulation and gender. J. Affect. Disord. 369, 489–498. doi: 10.1016/j.jad.2024.10.041, PMID: 39395680

[ref55] Zhou C. Yue X. D. Zhang X. Shangguan F. Zhang X. Y. (2021). Self-efficacy and mental health problems during COVID-19 pandemic: a multiple mediation model based on the health belief model. Personal. Individ. Differ. 179:110893. doi: 10.1016/j.paid.2021.110893, PMID: 36540084 PMC9756413

